# History of surgery at the University of Vienna: The three surgical schools

**DOI:** 10.1007/s00508-023-02318-w

**Published:** 2024-01-25

**Authors:** Laurenz Wolner, Paula Höfinger, Ernst Wolner

**Affiliations:** Medical University of Vienna, Medical University of Vienna, Vienna, Austria

**Keywords:** Education, Hospitals, Specialities, History of medicine, Austria

## Abstract

There are several publications on the history of surgery at the University of Vienna. None of these publications, however, sought to group the rich history of surgery in Vienna into distinct “surgical schools” of which, upon closer inspection, only 3 have emerged over the last 250 years. The oldest school dates back to Ferdinand Joseph von Leber and Vinzenz von Kern in the eighteenth and nineteenth centuries, the second school was founded by Theodor Billroth in 1867 and his student Anton Eiselsberg in 1900. The third school dates back to Jan Navratil, who was called to Vienna from Brno in 1967. Each of these schools is unique in that it maintained a degree of coherence, as knowledge, methodology and scientific focus were passed down in a chain of succession. These three schools also significantly influenced the rest of surgery in Vienna because most of the department chairs at the Vienna city hospitals or private hospitals were trained in one of these three schools.

## Introduction

There exist numerous publications on the history of Viennese surgery, exemplary here are the publications of Erna Lesky [1] and the comprehensive chronologically organized monograph of Karl Heinz Tragl called “Chronicles of Viennese hospitals” (“Chroniken der Wiener Krankenanstalten”) [2]; however, none of these publications considered the repeating patterns of succession, individual, and sometimes long-lasting influence of leaders in the field and topical coherence, which, in our opinion warrant that the history of surgery in Vienna be understood not as one single steady line of successions from one clinic head to the next through the centuries but rather as a series of different, distinguishable “surgical schools”. Defining and describing these schools, their emergence and influence, is the aim of this review. The history of the surgical subdisciplines, such as orthopedics, urology, neurosurgery and trauma surgery, is closely linked to the development of the surgical schools described but is not discussed in this paper, as each of these subjects would justify its own publication. Notably, while Austria’s first female doctor, Gabriele Possanner von Ehrenthal received her doctor’s degree in 1893 from the University of Geneva and 1897 from the University of Vienna, to this date, women have been and continue to be considerably underrepresented in Vienna’s surgical schools from 1900 onwards.

A surgical school within the meaning of this publication is best understood as a mean of organizing clinical training and the passage of knowledge at a university or clinical institution where the incoming successor is trained by his predecessor in the field. When analyzing the résumés of surgeons who have worked as surgical chairholders in Vienna, one will surprisingly find that the majority belong to one of three Viennese surgical schools, which were founded by Vinzenz von Kern in 1805, Theodor Billroth in 1867 and Jan Navratil in 1967. The influence of these schools cannot be overstated: up until 2010, all chairholders of the general, vascular, cardiothoracic, pediatric and plastic surgery departments were trained in one of these schools; similarly, the majority of the department chairholders from Vienna’s city and private hospitals received training in one of these schools [2].

It is generally accepted that surgery was considered a medieval handicraft in the Middle Ages and well into the eighteenth century. Surgery was organized in guilds, operated by masters who passed on their knowledge to their apprentices [3]. Although the University of Vienna was founded in 1365, structured theoretical teaching of surgery at the University of Vienna did not start until Ferdinand Joseph von Leber (1729–1808) [4]. Von Leber, originally an obstetrician, was largely supported by the famous reformer of the Austrian healthcare system, Gerard van Swieten. Alongside his teaching activities, von Leber was also a torture doctor, meaning he had to examine the fitness of delinquents before they were subjected to torture under the applicable penal laws. A stark advocate of more humane forms of punishment, he provided critical support to Joseph von Sonnenfels (1733–1817), a progressive writer, journalist, lawyer and politician, in his quest for the abolition of torture, which Maria Theresa ultimately banned by decree in 1776. Von Leber went on to become the personal surgeon of Maria Theresa [1].

At that time, the only connection between the university and the craft businesses was that the craft masters had to demonstrate certain anatomical knowledge, which was then examined by doctors of medicine at the Medical University. Surgery was recognized as an academic degree at the University in 1805; however, graduates were awarded the inferior title of “Doctor Chirurgicus”, with a shorter training period. The education was offered at the Josephinum, as well as at the University of Vienna.

This only changed with the university reform of 1849, when surgeons became doctors in the sense of a Dr. med as well [1].

Surgery was officially introduced as an academic subject at the University of Vienna in 1805 even though, as mentioned above, a few professors, Leber most importantly, had previously included surgery in their curriculum. There were two factors which in 1805 put surgery in the spotlight and established it as an academic subject, namely 1) increasing international influence and a developing surgical academia in France and England, and 2) the competition from the Josephinum founded in 1785 by Joseph II.

Joseph II was convinced that military doctors should be provided with proper training and education to ensure better care for wounded soldiers in the field. He commissioned his personal physician Giovanni Alessandro Brambilla to establish an institute that could provide such education. Subsequently, in 1785 the Josephinum was opened with all the rights of a university in the building that still exists today in Vienna’s ninth district [5]. At the same time, the Garnison Hospital was built as a teaching hospital in close distance to the Josephinum. As a counterweight to this influential institution promoted by The Emperor, the university ultimately increased the focus on surgery, which, for obvious reasons, formed one of the key subjects taught at the Josephinum.

## Results

### The first Viennese surgical school (1805–1932)

The founder of the first Viennese surgical school was Vinzenz von Kern (1760–1828). Von Kern received his training in Vienna with von Leber. Afterwards, he worked for 8 years as a senior surgeon at the Medical Lyceum in Ljubljana and in 1805, he took over the newly created chair for practical surgery at the University of Vienna [6]. Vienna owes two great achievements to von Kern. Firstly, he established a large surgical library for the further education of doctors. Secondly, he founded the surgical institute in 1807, 3 years after Gerhard von Vering (1755–1823) established such an institute at the Josephinum in 1804 [6]. Many surgeons were trained at the institute in 2‑year courses and went on to apply their newly found knowledge in practice and to provide surgical healthcare for the general population. Von Kern is also famous for improving the treatment of wounds and questioning traditional methods of wound treatment. Before von Kern, common wisdom was that wounds should always heal in pus. Von Kern, however, was able to show that primary healing could be achieved more efficiently with a continuous flush of water [7]. A practitioner as much as a teacher, von Kern was also one of the last bladder stone cutters. He favored the lateral stone incision according to Pajola over the previously often used perineal approach and, for his period of time, achieved sensational results with a mortality rate of only 25%, much lower than what other treatment options were able to provide [7]; however, even during his time stone cutting was replaced by lithotripsy [8].

Von Kern was succeeded by his student Josef von Wattmann (1798–1866). Von Wattmann is considered to be the founder of plastic surgery in Vienna, but also conducted experimental studies, such as studies on air embolism [9]. Above all, he was an outstanding teacher and had a great influence on the development of surgery at the University of Vienna. In 1848, as a result of the turmoil of the student revolution, he, like many other professors, was removed from his position and was unable to return. He remained highly respected, was ennobled, and had a large private practice and various public and political responsibilities [1]. The revolution of 1848 not only led to profound changes in the monarchy (Franz Joseph I was enthroned as the new Emperor) but also brought a fundamental university reform, including the establishment of two surgical university clinics. These two clinics determined the course of surgery in Vienna for the next 150 years [10].

The first head of the First Surgical University Clinic was von Wattmann’s student Johann Dumreicher (1815–1880). Dumreicher was primarily interested in orthopedics, and the later world-famous orthopedic surgeon Adolf Lorenz began his surgical career under him. Dumreicher was in constant conflict with his major rival at the second surgical university clinic, Theodor Billroth [1, 11]. Dumreicher accused Billroth of doing too little for the education of students and prioritizing research over education. The conflict with Billroth reached its peak when Dumreicher managed to get his student Eduard Albert appointed as his successor rather than Billroth’s student Vincenz Czerny, who later became head of the clinic in Heidelberg. Hence, the next head of the First Surgical University Clinic was Eduard Albert (1841–1900). Albert felt as a Czech for all his life, he was a talented writer and lobbied for a Czech nation [12]. Surgically, he was particularly interested in orthopedics and was one of the first to perform arthrodesis [13, 14]. At his clinic, Adolf Lorenz was able to develop into the world-famous orthopedist he would later become [15].

**Fig. 1:**
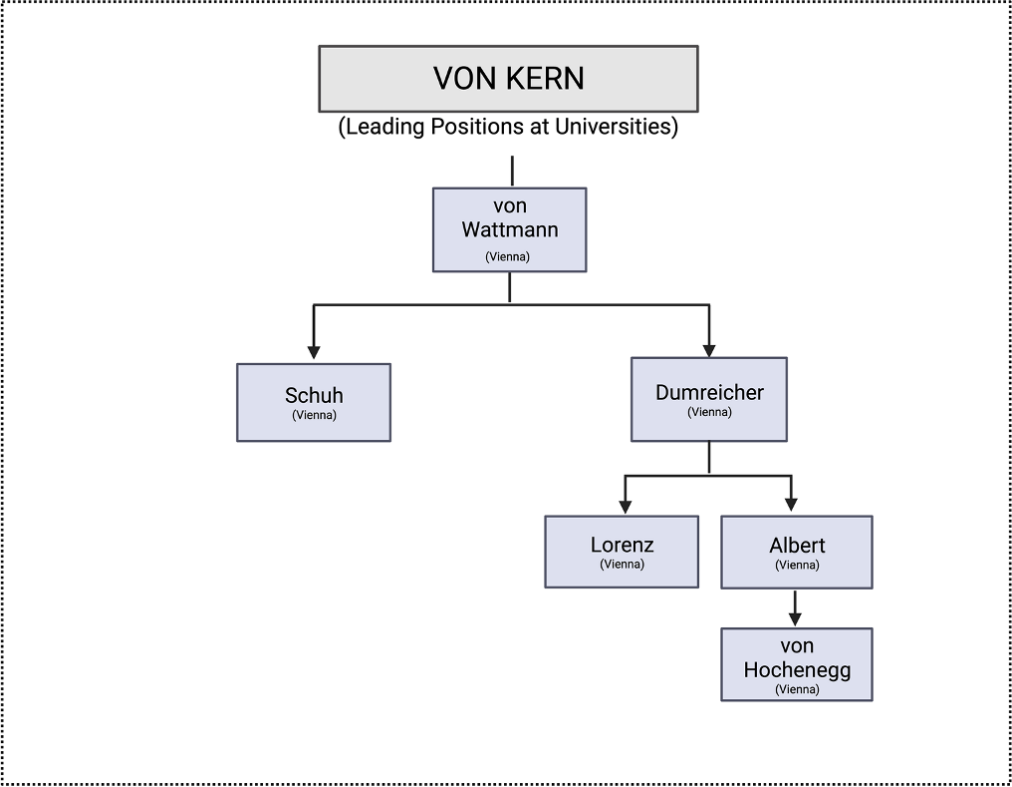
Leading positions at universities held by von Kern’s students (created with BioRender.com, based on Lesky [1], Killian [4])

With Albert, the time of chiefs of the First Surgical University Clinic which were trained in the first Vienna surgical school, ends (Fig. 1). Nevertheless, Albert’s student Julius von Hochenegg (1859–1940) was appointed head of the Second Surgical University Clinic. Hochenegg was chief physician at the Vienna Polyclinic before his appointment to the university. He was one of the most important colon surgeons of his time, and his methods included the sphincter-saving deep rectal cancer resection [16]. He opened a trauma department at the clinic. There, Lorenz Böhler, the great pioneer of trauma surgery, began his surgical training [6].

Franz Schuh (1804–1865) also belonged to the first Vienna surgical school. He was the first head of the Second Surgical University Clinic. Schuh was a student of von Wattmann. He first completed a surgical education to become a Dr. chir. and then returned to the university and obtained his Dr. med. He was the first surgeon in the monarchy to view surgery not only as a practical subject but also as a science. He conducted various experimental studies and together with his congenial partner, the internist Joseph Skoda, he was able to diagnose and puncture pleural and pericardial effusions through percussion and auscultation [17]. In 1846, he was the first in the monarchy to introduce ether anesthesia, which had been described for the first time in Boston a short time before [6].

### The second Viennese surgical school

**Fig. 2:**
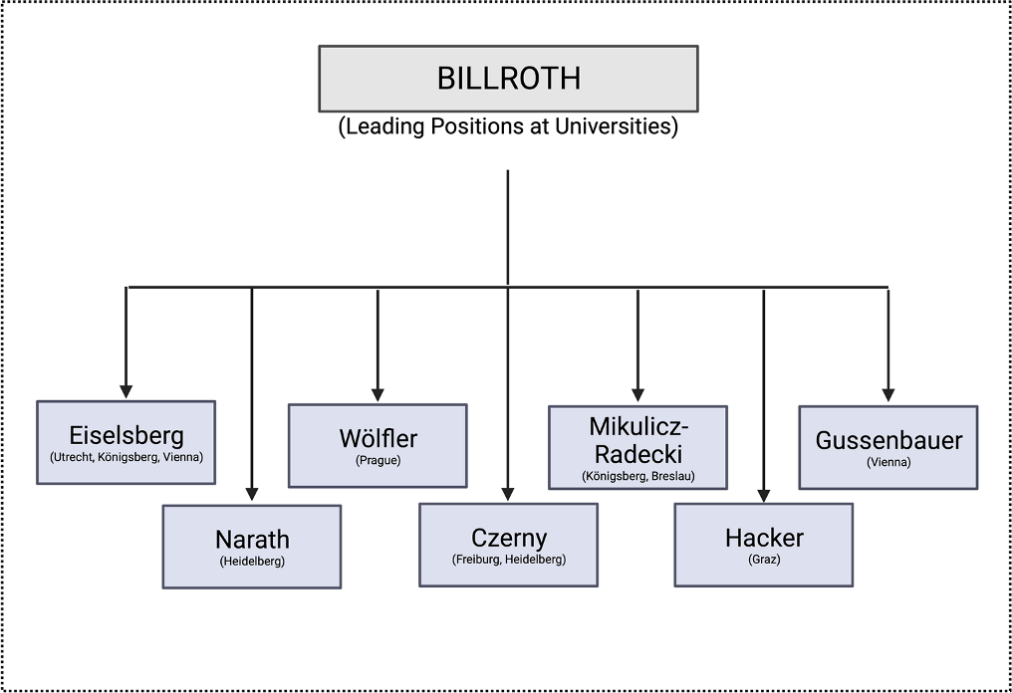
Leading positions at universities held by Billroth’s students (created with BioRender.com, based on Lesky [1], Killian [4])

After the unexpected and sudden death of Schuh, the faculty, particularly driven by the pathologist Rokitansky and the physiologist Brücke, decided to appoint a Prussian, Theodor Billroth (1829–1894), as head of the Second Surgical University Clinic. This is surprising, given that Austria had recently suffered a decisive military defeat against Prussia at Königgrätz. Billroth received his training from Langenbeck in Berlin, was a Professor in Zurich at 30 years old, and was appointed to Vienna at the age of 37 years. Billroth became one of the most influential surgeons in the second half of the nineteenth century. Various surgeries that he performed for the first time, especially gastrectomy, which still bears his name, made him famous worldwide. Besides, Billroth was also a great researcher. Unlike his counterpart at the first surgical university clinic, Dummreicher, he believed that students should not only receive practical training, but also an understanding of science and research. Furthermore, he engaged in many public affairs, was a co-founder of the Vienna ambulance service, founded his own hospital for a better training of nursing staff, the Rudolfinerhaus, was a member of the Reichsrat, co-founder of the Medical Association, and created a dedicated event space for physicians, today the Billrothhaus in the 9th district of Vienna [2, 18]. Above all, however, he was the founder of a large surgical school, which, particularly through his student Eiselsberg, continues to have an impact in Austria to this day. Of his students, seven were appointed as clinic chairholders at universities in German-speaking countries (Fig. 2). Billroth, a German nationalist, was certainly no friend of Jews and has repeatedly been associated with antisemitism. In his famous book on the teaching and learning of medical sciences, a number of antisemitic remarks can be found [19]. Noteworthy, his attacks were primarily directed against the Jewish students who had immigrated from the eastern parts of the monarchy, accusing them of lack of diligence and insufficient attendance of lectures [19].

In this respect, however, Billroth also remains a figure of some ambiguity even though his view on Jewish doctors was ostensibly a racial one. He was an honorary member of the Association to Combat Anti-Semitism and stated the following:

“I do not wish being brought together with people who find themselves popular in railing against Jews. Therefore, I do not hold back my experiences concerning these issues. Jews on the one hand, are talented for natural science and the medical profession on the basis of their vivid imagination and on the other hand, they will be successful in their work because of their sharp reasoning, their energy and endurance even when their material resources are limited.” [20, p. 152]

Undoubtedly, the most influential student of Billroth was Anton von Eiselsberg (1860–1939). He was awarded a habilitation at the University of Vienna in 1890 for a work on tetany and was later appointed head of the First Surgical University Clinic in Vienna after short periods in Utrecht (1893) and Königsberg (1896) [1, 2]. Together with other students of Billroth he worked untiringly on further developing gastrectomy and introduced various modifications. In addition, he was particularly interested in neurosurgery and trauma surgery [21]. Eiselsberg was highly respected by his patients and had a large international clientele. Furthermore, he was an excellent academic teacher, no less than nine of his students were appointed as chairs at various European universities, not to mention the many other assistants who received positions as surgical chairholders in non-university hospitals. Eiselsberg died in 1939 shortly after his retirement in a train accident in Upper Austria, near to his place of birth [22].

**Fig. 3:**
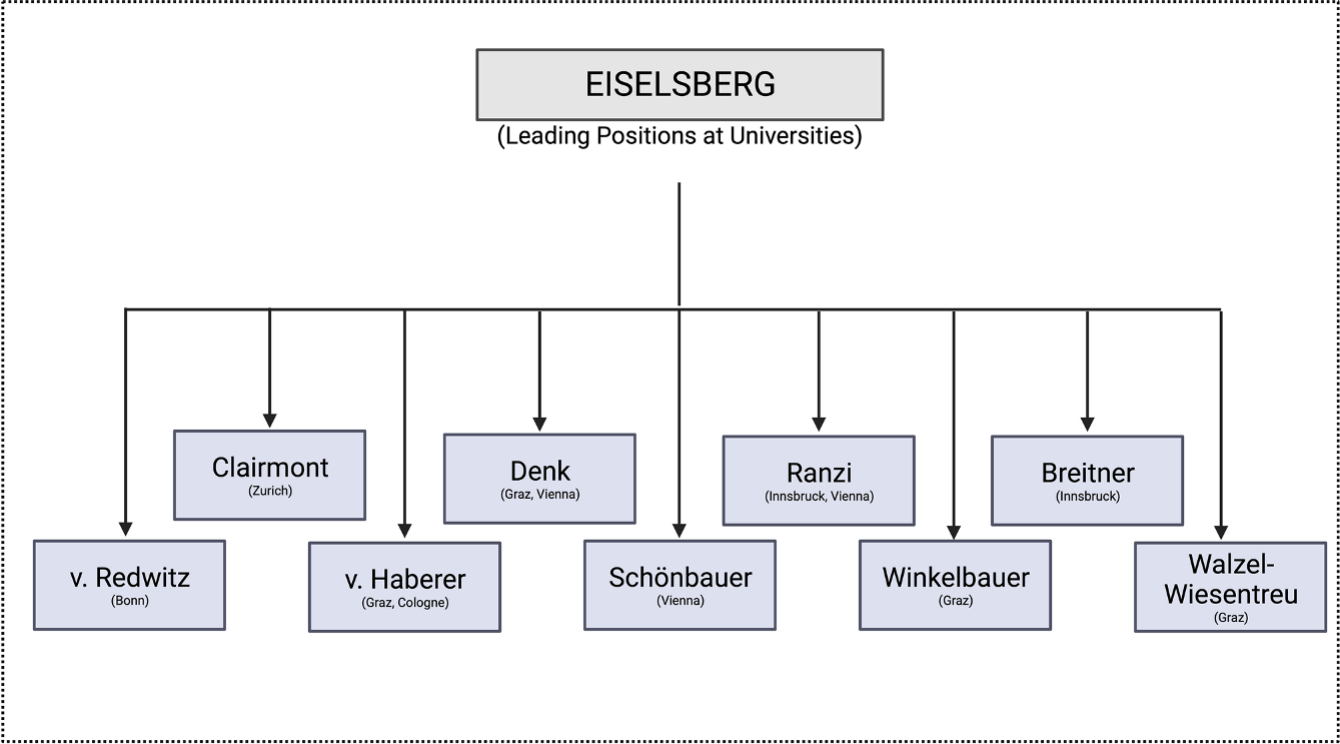
Leading positions at Universities held by Eiselsberg’s students (created with BioRender.com, based on Lesky [1], Killian [4])

The fact that all subsequent heads of the First Surgical University Clinic can be referred to as students or “grand students” of the Billroth-Eiselsberg era has shaped Viennese surgery for years to come (Fig. 3).

Egon Ranzi succeeded Eiselsberg at the First Surgical University Clinic. After working as a chief physician at the Rudolfstiftung in Vienna and after being surgical chair at the University of Innsbruck, he was called to Vienna in 1932. Ranzi was also politically involved in the Dolfuß-Schuschnigg regime and was forcibly retired in 1939. He died shortly afterwards from a kidney disease [23, 24].

His successor was Leopold Schönbauer (1888–1963). Schönbauer was also a student of Eiselsberg and was a talented general surgeon with a special interest in neurosurgery, where he further educated himself by learning from the famous American neurosurgeon Cushing. Like many members of the faculty at the University of Vienna, he was a member of the National Socialist German Worker’s Party (Nationalsozialistische Deutsche Arbeiterpartei [NSDAP]) and a functionary in the medical system of Nazi Germany. Schönbauer was never properly denazified and his efforts in relation to preventing the destruction of the Vienna General Hospital in 1945 ultimately allowed him to retain his position as head of the clinic, despite formal denazification proceedings being instituted in 1946. He later went on to become both the university rector and a member of the National Council [25].

Schönbauer had a huge influence on the structural development of the clinic; some of the units had almost 400 beds. After his retirement in 1960, clinics for urology, orthopedics, neurosurgery and maxillofacial surgery were separated from his clinic [26]. The separation of medical physics took longer. The head, Hans Heinrich Jantsch (1918–1994) only attained the position of associate professor in 1971, and in 1974 an independent unit was created.

Paul Fuchsig (1908–1977) was Schönbauer’s successor. Fuchsig began his surgical training with Egon Ranzi in Innsbruck, followed him to Vienna, and continued under Schönbauer. In 1957, he became chief physician at the Elisabeth Hospital in Vienna and was appointed head of the First Surgical University Clinic in 1961. Thyroid surgery was Fuchsig’s specialty. He was the first in Vienna to establish a department of experimental surgery and, together with anesthesia, a surgical intensive care unit [27]. During his time, the field of trauma surgery became independent through the establishment of two professorships. Additionally, plastic surgery (Hanno Milesi 1927–2017) and vascular surgery (Franz Piza 1925–2016) were strengthened through departmental professorships.

The last head of the First Surgical University Clinic before its move to the new general hospital was Arnulf Fritsch (1926–2014). Fritsch started his surgical training at the Franz Josef Hospital in Vienna, then switched to Fuchsig at the clinic. In 1974, he was appointed chief of the surgical department at the Floridsdorf Hospital, and in 1977 he was appointed head of the First Surgical University Clinic. Fritsch was a talented general surgeon with a special interest in the surgery of the bile ducts, liver, and pancreas [28]. During his tenure, the fields of kidney and liver transplantation were considerably strengthened [2, 29].

The influence of the Billroth-Eiselsberg school was not limited to the First Surgical Clinic. In 1931, after Hochenegg, the Eiselsberg student Wolfgang Denk (1882–1970) was appointed head of the Second Surgical University Clinic. Denk was already very interested in thoracic surgery at an early stage, and in 1912 he published an experimental paper on the removal of the esophagus [30]. In 1924 he became chairholder at the Rudolfstiftung, in 1928 he was appointed head at the University of Graz, and in 1931 he became head of the Second Surgical University Clinic in Vienna. Despite being politically active in the Dolfuß-Schuschnigg regime, he was able to retain his office during the Nazi regime and was rector from 1948–1949. In 1957, he was a candidate in the presidential election, which he lost. Denk is considered to be one of the founders of thoracic surgery in Austria, with significant support from his student Georg Salzer (1903–1995). He was also active in the field of cancer research and a major contributor to the formation of the Cancer Research Institute in Vienna [31]. He was president of the College of Physicians in Vienna and of the Supreme Sanitary Council [31]. Furthermore, Denk made an important contribution to the development of anesthesia in Austria, when, in 1947 he sent the young assistant Otto Mayrhofer (born 1920) to London to learn about intubation anesthesia. Mayrhofer later became the first clinic head for anesthesia in Vienna.

After Wolfgang Denk’s retirement, the faculty struggled to agree on a successor [32]. Finally, it was Denk’s student, Hubert Kunz (1885–1979), who took over. Kunz started his training in Vienna with Eiselsberg and Ranzi and then switched to Denk, first in Graz and then in Vienna. He then became chief at Wilhelminen Hospital and later at Lainz Hospital. Finally, he was appointed head of the Second Surgical University Clinic in 1957. Kunz was a general surgeon, with a main interest in emergency surgery. In 1948, he published his most important work, a book on surgery of acute abdomen [33]. Immediately after his appointment, he campaigned for the renovation of the clinic built in 1904 and achieved a complete redesign, including an intensive care unit with 16 beds. The beginning of heart surgery in Vienna also falls into his tenure [34]; however, the faculty was apparently dissatisfied with the progress of, among other fields, heart surgery at the clinic. In the absence of a suitable candidate from in-house, after Kunz’s retirement (1967), an appointment from outside was considered. This eventually led to the establishment of the third Viennese surgical school.

### The third Viennese surgical school

After only short negotiations, the faculty council agreed to appoint Jan Navratil (1909–1992) as the new head of the Second Surgical University Clinic. Navratil, who at that time was the head of the Second Surgical University Clinic in Brno, was undoubtedly the leading heart surgeon in the entire communist east at the time. Navratil was a universally trained surgeon. After World War II, he established a department of urology and later neurosurgery at the university clinic in Brno. After his appointment as clinic head in Brno, he turned to heart surgery. In 1956, he was one of the first in Central Europe to perform open heart surgery in deep hypothermia [35]. The first cardiac operation with a heart-lung machine was performed 2 years later in Germany and Navratil was quick to perform such an operation in Brno as well [36]. As the communist states of the Eastern Bloc were largely excluded from western technology, he had to make his own heart-lung machine, artificial heart valves and vascular prostheses. As a result, he had an enormous amount of experience in surgical research. At his inaugural lecture in 1967 in Vienna on the future of heart surgery, he presented experiments on dogs with transplanted hearts and mechanical pumps [37]. After his appointment he pushed for the introduction of coronary surgery and aneurysm surgery in Vienna. He also established the departments for pediatric surgery (Fritz Helmer 1922–2009) and plastic surgery (Gerhard Freilinger 1928–2022).

Above everything else, his utmost passion was surgical research, particularly mechanical circulatory support. In 1969, he was the first in Europe to implant an intra-aortic balloon pump in a human [38]. He established a large research department on the roof of the clinic, which, in terms of research topics, to this day is in part reflected in the focus areas of the Institute for Biomedical Research and Translational Surgery in the new Vienna General Hospital today. To financially ensure the continuation of research, especially the artificial heart research, he contributed to the establishment of the Ludwig Boltzmann Institute for Heart Surgery Research [39]. The Navratil School’s great research affinity is also reflected in the establishment of the departments for biomedical technology, experimental surgery, now biomedicine and biostatistics, which were later upgraded by the faculty with professorships and staff from the clinic (Thoma, Losert, Podesser, Schreiner). Navratil was a very modest person and a great supporter and promoter of young employees. He retired in 1980 and left behind a large number of students who would later achieve leading positions.

**Fig. 4:**
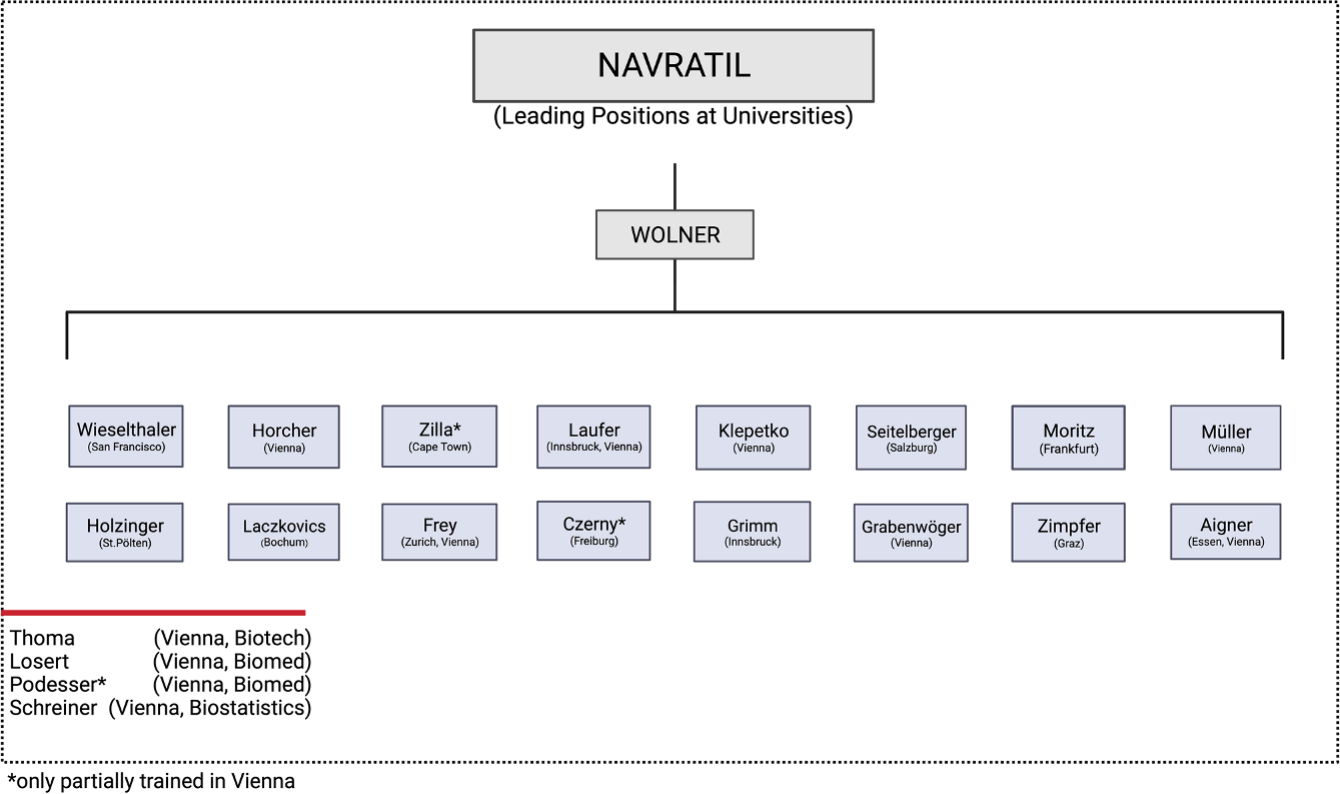
Leading positions at Universities held by Navratil’s and Wolner’s students (created with BioRender.com, based on Lesky [1], Killian [4])

Ernst Wolner, a student of Jan Navratil, succeeded him as the last head of the second surgical university clinic. During his tenure, Vienna saw its first heart transplantation and first lung transplantation, and a large transplant center was established [40, 41]. The artificial heart program was introduced and constantly developed with a rotary pump being successfully implanted for the first time worldwide [42]. At the same time, the clinic became more internationally renowned and several surgeons were appointed to foreign universities. There is also a substantial number of employees of the third Viennese surgical school, who for the most part received their entire medical education in Vienna and were subsequently appointed to leading positions at universities and other medical institutions around the world (Fig. 4).

In 1994, with the move to the new Vienna General Hospital, a complete restructuring of the university clinic for surgery was implemented. As a result, six separate clinical departments were established. In this context it is interesting that three of the six departments were staffed by employees of the old First Surgical University Clinic, and three by employees of the Second Surgical University Clinic [2].

**Fig. 5:**
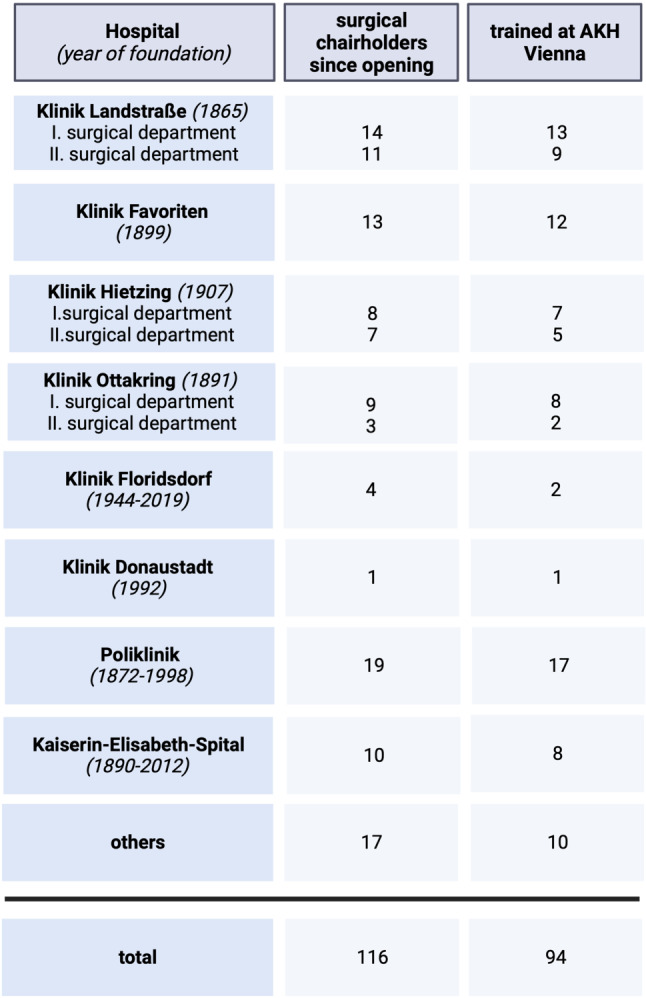
Number of chairholders at nonuniversity surgical departments in Vienna (created with BioRender.com, based on Lesky [1], Killian [4]) AKH = Allgemeines Krankenhaus (General Hospital)

Surgery in Vienna, however, is not limited to the Vienna General Hospital and is also performed in a number of city hospitals, clerical hospitals and private clinics. In Fig. 5, the heads of the most important non-university surgical departments in Vienna since their opening are listed only by their number, indicating whether they received their surgical training at the Vienna General Hospital. As can be seen from (Fig. 5), 94 of the 116 surgical heads of Vienna’s hospitals (since their foundation) were trained at the two university clinics, which demonstrates the enormous importance of the surgical university clinics for the surgical education and the high standard of surgery in all of Vienna’s hospitals [2].

## Discussion

The history of surgery in Vienna from the early nineteenth century onwards is multifacetted and characterized by a steady increase in the importance of the field of surgery in the medical profession as well as a constant development towards higher standards in surgery, new insights and methods discovered by relentless research and, as consequence, an ever-increasing specialization and differentiation into distinct subdisciplines. Several interesting structural elements and repeat patterns can be observed when looking at this history from the perspective of different schools of surgery, of which, in our opinion, 3 existed from 1805 until 1994.

First, it is fair to say that the emergence of the three surgical schools is, to a considerable extent, due to the fact that succession into leadership positions occurred predominantly from in-house, i.e., leadership positions were almost exclusively taken over by students of the outgoing head of the clinic.

Second, when this precedent was broken, which occurred only twice in 200 years, namely when in 1867 Billroth was appointed and in 1967 Navratil, this resulted in a substantial acceleration of the development of the respective schools and ultimately a steep increase in enhancing the profile of surgery in Vienna overall. This does not mean to imply that in-house succession was a hindrance to the development of the discipline and, as mentioned above, e.g., in relation to Mayrhofer, knowledge from outside and other countries was carefully implemented by the more circumspect heads of the clinic. It was, ultimately, a combination of breaking with long-standing traditions and finding the right individuals for doing so, which helped the second and third surgical schools in Vienna to thrive.

Third, in terms of appointments to chairs at domestic and foreign universities the Billroth-Eiselsberg school per se (Figs. 2 and 3) and the Navratil school (Fig. 4) were most successful and hence most influential on a broader scale beyond merely the University of Vienna. Unrelated to that, it is interesting to observe that before World War II, it was not unusual that doctors working in Viennese municipal hospitals, often the respective heads of the clinic, were later appointed as heads of the clinics in Graz and Innsbruck. After the end of the war, this only happened rarely (e.g., Paul Huber, Innsbruck). Also, historically, several heads of clinics (Gusenbauer, Hochenegg, Denk, Ranzi, Kunz, Schönbauer, Fuchsig, Fritsch) served at a Vienna municipal hospital before their appointment to the University of Vienna. Today, this is exceedingly rare because, on average, municipal hospitals cannot compete with university clinics when it comes to research.

In general, heads of clinics should serve with empathy, dedication, knowledge, and openness to the new aspects of surgery as a craft, art, and science. Tradition is, as they say, the preservation of the past in the sense of passing on the fire and not admiring the ashes. Perhaps not all heads of clinics mentioned here have lived up to these ideals to the same extent, but many have contributed in different ways to take their skills and knowledge beyond the local context. It will continue to be the responsibility of the relevant university committees to find surgeons for leadership positions who can maintain the reputation of surgery and the university both internally and externally.
